# Medical Quacks

**Published:** 1886-09

**Authors:** 


### *MEDICAL QUACKS.*

There are *quacks* in all professions and employments; quack ministers, doctors, lawyers, dentists, but the most pestiferous among them is the *quack doctor.* He has only to impose himself upon a community, put out a sign with the prefix of Dr., to find patrons, many of whom become in a manner his victims. Without medical training, and ignorant alike of the human anatomy and the properties of the remedies he assumes to administer, he holds himself out to a confiding public as fit to be intrusted with the treatment of the most delicate and dangerous maladies, which he is always ready to undertake with all the assurance of a skilful practitioner. In cases of great emergency, when the physician nearest at hand is likely to be called, irrespective of family preferences, the *quack* stands an even chance with his betters, and it is on just such occasions that he finds his way to the bedside of patients who trust him, simply because they do not know him, and continue to trust him till they find him out.

In all our large cities we have a class of crooked operators whose endeavor to ingratiate themselves into the confidence of the innocent and unwary, in order to despoil them of their valuables. These are called "confidence men," and it is of daily occurrence that some of our friends from the country have cause to regret a too hasty acquaintance with them. The quack doctor is a confidence man of the meanest type. He deliberately sets to work to gain the confidence of his patrons by the most barren false pretenses, well knowing that were his true character understood no house would receive him. Is there no punishment for this? To a certain extent there is, but the difficulty with the medical laws in general is that the object aimed at has been the protection of a privileged class, rather than of the people at large. They go too far in one way and stop too short in another.

While it will be readily enough conceded that no one should be allowed to practice either surgery or medicine without the requisite qualifications, it is also well known that many individuals are naturally gifted with healing powers and capable of affecting cures, without the use of medicines, and who never resort to their use. Of this class we are able to call to mind many notable examples. But as a rule they are not *Doctors* in any true sense, and have no right to the prefix of "Dr." to their names, which, as now indiscriminately used, is just as likely to signify the veriest pretender as otherwise. There is a simple and sure remedy for this to be found in wholesome legislation. In New York it is a misdemeanor punishable by fine for one in active business *solus* to append to his name the words or characters "& Co.," as calculated to mislead. Now let the law take a like step in the direction indicated, and after defining what "Dr." shall signify, make it a penal offense for anyone to prefix or affix it to his sign or business card without legal right. This would cause no little commotion amongst a multitude of small tin signs in this city alone, and not a few lazy, lubberly ignoramuses who thrive upon the credulity of their unquestioning patrons, would find their occupation gone.

The late disclosures which have been made in the notorious Robinson poisoning case furnish us with a case in point, in the instance of one self-styled Dr. Beers, who, whilst serving out a sentence in a Connecticut prison, managed to pick up a little knowledge of medical terms, and when at length the doors were thrown open to him, he assumed the role and title of Doctor, and set himself up as a specialist for the cure of intemperance and the opium habit. Among others, the cities of New York and Boston were selected as the theatre of this versatile doctor's exploits. Had he a diploma? Not *he*. How could he have? Was he not at once the founder, dean and faculty of his own school? It had been no more absurd to demand of Adam a marriage license, or of Cain an acquittal by a jury of his countrymen.

Dr. Beers was a specialist, and the only one in the wide world in his peculiar methods. His way of curing bad habits was to indulge them —to *surfeit them*. The opium eater took his pills and escaped the gnawing, teasing, devouring sensation of other remedies. At last he had found his Mecca and was blessed, and so was Dr. Beers till the secret came out. It was very simple. The pills themselves were *opium*.

Thus it was that the Doctor improved upon the Homeopathic idea as expressed in its well-known maxim, "Similia, similibus curantur." This time it is not alone the "hair of the dog," but the whole animal. We understand the Doctor has been able to persuade the State to renew its hospitalities to him—*gone back for another course.*

